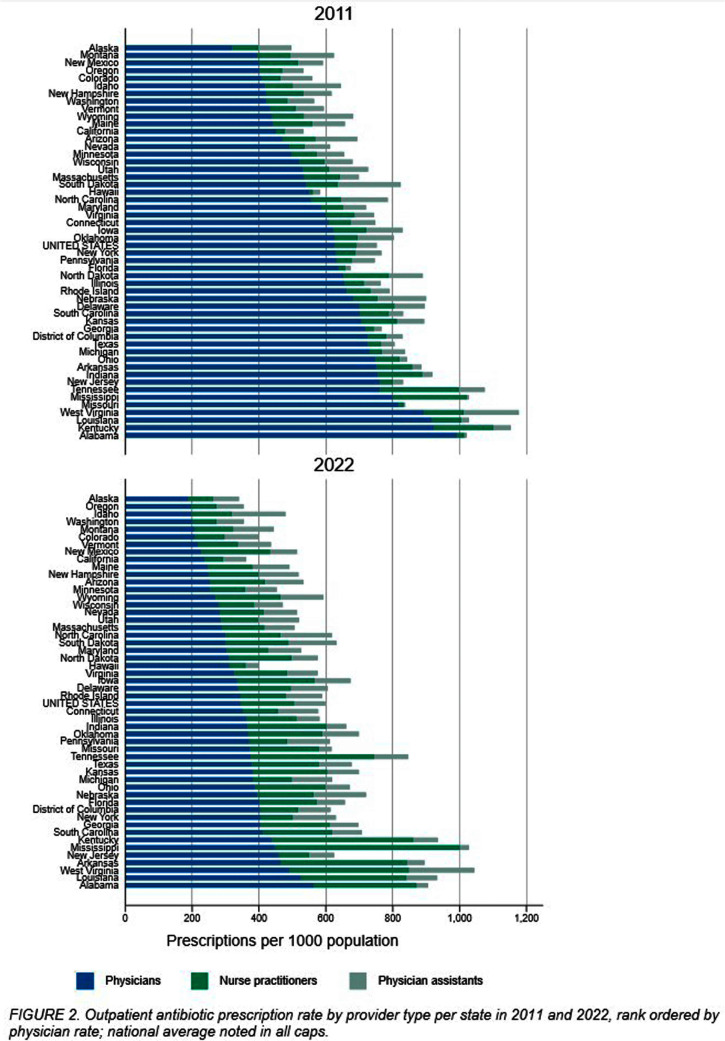# Changes in outpatient antibiotic prescriptions by U.S. physicians and advanced practice providers, 2011 and 2022

**DOI:** 10.1017/ash.2024.122

**Published:** 2024-09-16

**Authors:** Mohsin Ali, Guillermo Sanchez, Katryna Gouin, Adam Hersh, Sarah Kabbani

**Affiliations:** Centers for Disease Control and Prevention (CDC); University of Utah

## Abstract

**Background:** The number of advanced practice providers (APPs)—nurse practitioners (NPs) and physician assistants (PAs)—continues to expand across the United States. Several studies suggest differences in antibiotic prescribing rates and appropriateness by APPs compared to physicians. The objective of this analysis is to characterize population- and provider-specific outpatient antibiotic prescribing rates among physicians and APPs nationally, by state, and within urban versus rural counties. **Methods:** We estimated outpatient oral antibiotic prescription rates for 2011 and 2022 using county-level prescription dispensing data from IQVIA Xponent® (numerator) and population census estimates (denominator). Provider specialty denominators were provided by IQVIA, based on data from the American Medical Association. Counties were classified as urban or rural per the 2013 National Center for Health Statistics classification. National and state-level prescription volume, rates per 1000 population, and average number of prescriptions per provider were calculated for physicians, NPs, and PAs. We assessed the degree to which provider-specific rates explained the variance of the overall rate by state, using the coefficient of determination (r2) from Pearson’s correlation. **Results:** Between 2011 and 2022, overall U.S. antibiotic prescribing declined from 877 to 709 per 1000 population, a 19.2% relative reduction. The provider-specific proportion of the overall prescribing rate relatively decreased by 32% for physicians but increased by 157% for APPs (NPs 229%, PAs 86%; Figure 1). State-level antibiotic prescribing rates varied by provider type for both years, shifting towards proportionally greater APP prescribing in 2022 (Figure 2). For 2011 and 2022, physician prescribing rate strongly correlated with the overall state rate (r2 = 0.83 in 2011 versus 0.80 in 2022), whereas the correlation of the NP prescribing rate increased (r2 = 0.20 in 2011 versus 0.76 in 2022). A total of 60,327 (7.2%) physicians practiced in rural settings in contrast to 42,876 (12%) NPs and 14,495 (9.4%) PAs in 2022. Providers in rural counties prescribed more antibiotics per provider on average compared to urban counties; rural physicians prescribed 57% more antibiotics per provider (207 vs 132 antibiotics per provider), rural NPs prescribed 115% more (284 vs 132), and rural PAs prescribed 53% more (289 vs 189). **Conclusions:** The relative contribution of APPs to outpatient antibiotic prescriptions more than doubled over the past decade, accounting for 1 in 3 prescriptions in 2022. This contribution was especially prominent among NPs in rural counties. Further evaluation of antibiotic prescribing appropriateness among APPs and integration of APPs into antibiotic stewardship efforts in various settings.

**Figure:**
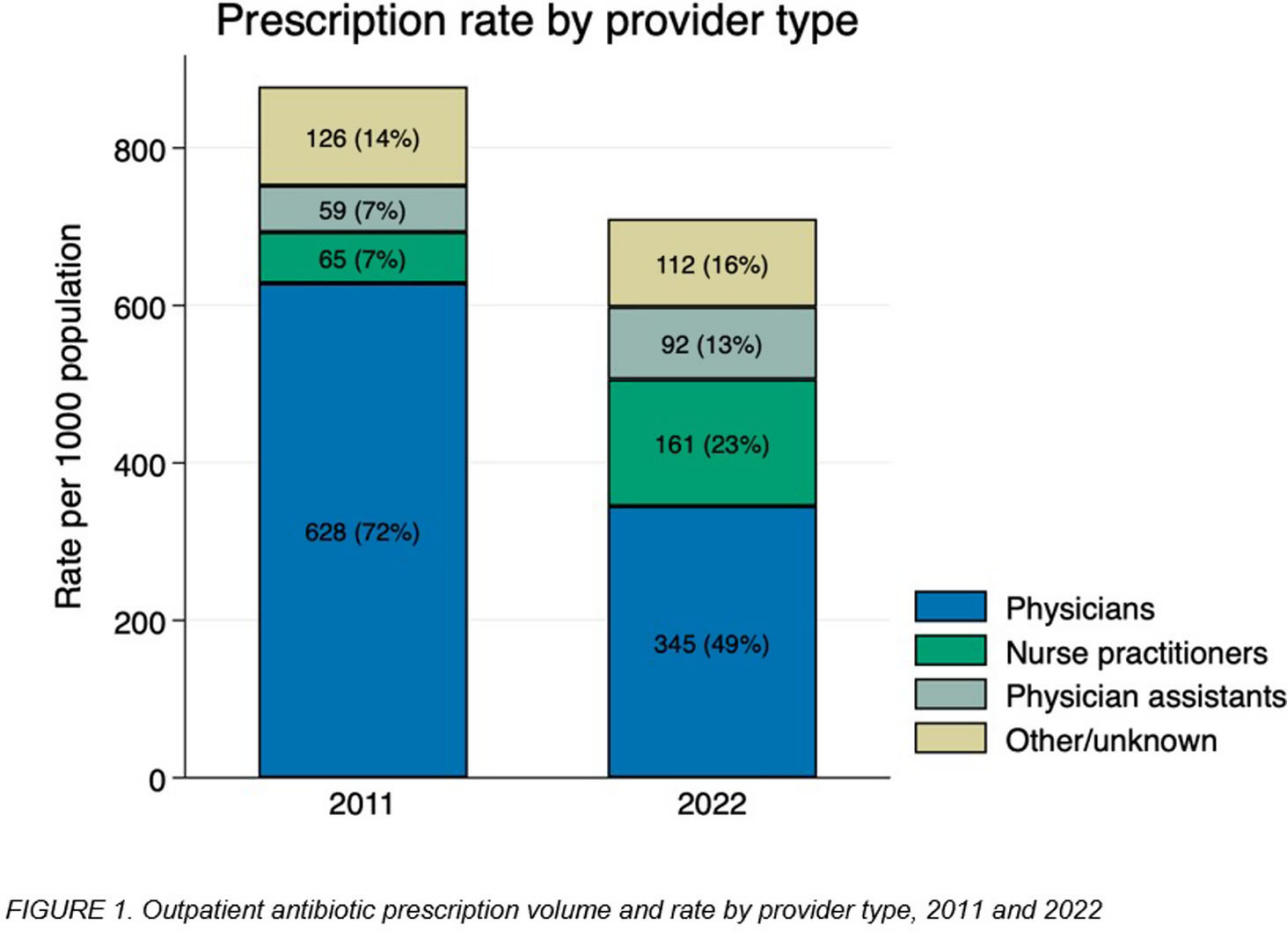

**Figure:**